# Orthoscape: a cytoscape application for grouping and visualization KEGG based gene networks by taxonomy and homology principles

**DOI:** 10.1186/s12859-016-1427-5

**Published:** 2017-01-27

**Authors:** Zakhar Sergeevich Mustafin, Sergey Alexandrovich Lashin, Yury Georgievich Matushkin, Konstantin Vladimirovich Gunbin, Dmitry Arkadievich Afonnikov

**Affiliations:** 1 Institute of Cytology and Genetics SB RAS, Lavrentiev Avenue 10, Novosibirsk, 630090 Russia; 2 Novosibirsk State University, Pirogova st. 2, Novosibirsk, 630090 Russia

**Keywords:** Cytoscape plugin, Ortholog, Paralog, Metabolic pathway, Gene regulatory network, Evolution, Phylostratigraphy, Evolution

## Abstract

**Background:**

There are many available software tools for visualization and analysis of biological networks. Among them, Cytoscape (http://cytoscape.org/) is one of the most comprehensive packages, with many plugins and applications which extends its functionality by providing analysis of protein-protein interaction, gene regulatory and gene co-expression networks, metabolic, signaling, neural as well as ecological-type networks including food webs, communities networks etc. Nevertheless, only three plugins tagged ‘network evolution’ found in Cytoscape official app store and in literature. We have developed a new Cytoscape 3.0 application Orthoscape aimed to facilitate evolutionary analysis of gene networks and visualize the results.

**Results:**

Orthoscape aids in analysis of evolutionary information available for gene sets and networks by highlighting: (1) the orthology relationships between genes; (2) the evolutionary origin of gene network components; (3) the evolutionary pressure mode (diversifying or stabilizing, negative or positive selection) of orthologous groups in general and/or branch-oriented mode. The distinctive feature of Orthoscape is the ability to control all data analysis steps via user-friendly interface.

**Conclusion:**

Orthoscape allows its users to analyze gene networks or separated gene sets in the context of evolution. At each step of data analysis, Orthoscape also provides for convenient visualization and data manipulation.

**Electronic supplementary material:**

The online version of this article (doi:10.1186/s12859-016-1427-5) contains supplementary material, which is available to authorized users.

## Background

Biological networks arise in completely all fields of modern biology gathering both ‘real’ (experimental data etc.) and virtual (modeling and simulation data) biological information [1–6]. There are software packages to work with biological networks with less or more biological specialization, availability and interactivity [7–11]. Among them, Cytoscape [7, 12] is one of the most comprehensive tools for performing all-round analysis of biological networks. There are many plugins which extend the functionality of Cytoscape by providing visualization and analysis of protein-protein interaction networks [13, 14], including PINA4MS (http://apps.cytoscape.org/apps/pina4ms), Strongest Path (http://apps.cytoscape.org/apps/strongestpath), gene regulatory [15, 16] and gene co-expression [17, 18], metabolic [18–20], signaling [21, 22] as well as ecological-type networks including food webs, communities network and others. In spite of such great diversity, evolution-oriented plugins are in short supply: just three plugins tagged ‘network evolution’ (even not ‘biological evolution’) in Cytoscape official app store and in literature. These include ANIMO [23], TieDIE, NetworkEvolution [24]) and a couple of plugins concerning orthology analysis (HOMECAT [25] and OrthoNets [26]).

KEGG (Kyoto Encyclopedia of Genes and Genomes, www.kegg.jp) is a set of databases containing biological information of various types, such as genes, genomes, protein interaction networks, pathway maps and many others. KEGG Pathway is a collection of manually curated maps representing the molecular interaction and reaction networks for various biological processes, such as metabolism, genetic information processing, human diseases, etc. Number of tools have been developed to operate with KEGG pathway maps including CyKEGGParser [27], ANIMO [28] and SIREN [16].

In this work, we have developed a Cytoscape application (plugin) Orthoscape aimed to analyze evolutionary information in the gene sets and networks: (1) the orthology relationships between genes; (2) the evolutionary origin of gene network components; (3) the evolutionary regime (diversifying or stabilizing, negative or positive selection) of orthologous groups in general and/or branch-oriented mode. See Additional file 1 for the Orthoscape jar-file and Additional file 2 for manual.

Clear evolutionary ideas underlying the Orthoscape application and its ability to control all data analysis steps via user-friendly interface will aid biologists in better understanding the factors of evolution and functioning of gene network.

## Methods

Orthoscape implemented in Java 1.8 language to use with Cytoscape 3.0 version or higher. Homology, taxonomy, protein domains data, as well as nucleotide and amino acid sequences are extracted from KEGG databases, hence, requiring Internet connection. All downloaded data could be stored in local Orthoscape database, which may require up to several GB hard drive space and later may both speed up the work and decrease dependence on connection to the KEGG.

### The orthoscape workflow

**Fig. 1:**
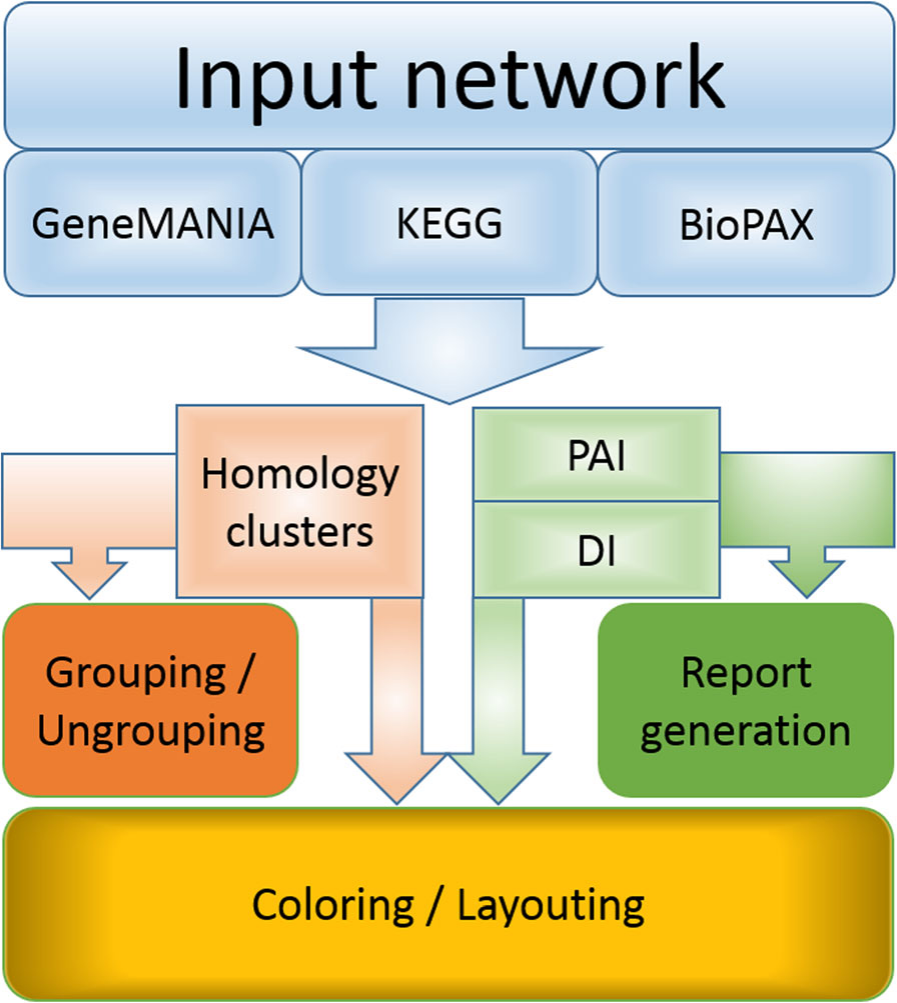
Principle diagram of the Orthoscape workflow

Figure 1 depicts a workflow of Orthoscape, with a gene network (or just a set of genes) used as an input which may be presented in several ways:

1. Using the CyKEGGParser plugin (http://apps.cytoscape.org/apps/cykeggparser), a network may be imported directly from the KEGG database. Once it is imported, it is ready to work with the Orthoscape.
2. Using the GeneMANIA plugin (http://apps.cytoscape.org/apps/genemania), a network may be reconstructed on the set of necessary genes. The obtained network should be converted using “Orthoscape - > convert GeneMANIA Network” option.
3. Using the CyPath2 plugin (http://apps.cytoscape.org/apps/cypath2) (CyPathwayCommons), the user may choose a network from the list of filtered networks (by presence of necessary genes) in BioPAX format. The obtained network should be converted using “Orthoscape - > convert BioPAX Network” option.

Then gene network is analyzed by the Orthoscape core, which performs the following tasks: (1) reconfiguration (layouting and grouping) of gene networks on the base of sequences homology; (2) coloring and grouping of genes on the base of evolutionary characteristics such as Phylostratigraphic Age Index (PAI) and Divergence Index (DI) (3) report generation. As a result, the information on genes, their interactions and evolutionary characteristics is represented in compact and convenient manner. It provides users a better understanding of gene networks structure and enables to perform analysis of network evolution.

## Implementation

### Analysis of homologous sequences

For each gene from a gene network (or gene set), the Orthoscape generates lists of potential homologs (paralogs in case of gene network and/or orthologs in case of set of genes from various genomes) according to SW-Score (Smith-Waterman score [29]) and identity values set up by the user. These operations are performed using requests to KEGG database via REST API protocol (http://www.kegg.jp/kegg/docs/keggapi.html). After that, Orthoscape joins genes into clusters using one of three possible types of similarity measures for the sequence comparison: (1) SW-Score; (2) identity value; (3) domain composition from the proteins previously filtered by SW-Score and/or identity.

The similarity according to domain composition could be calculated in either simple or detailed way. Simple one implies the analysis of domain frequencies among all potential filtered homologs. The user may set the ‘threshold’ number *T* of domains to be the same in gene and its potential homolog. At first, we construct the ordered list of most presented domains among all homologs found. If a homolog contains first T domains from this list, then it is accepted and the homologous genes join. The detailed analysis additionally allows user to choose the particular domains essential for him/her. If a potential homolog does not contain any of those domains, it is deleted from the list.

Once genes are joined into clusters according to their similarity, the Orthoscape allows the following additional analysis of the gene network: (1) group/ungroup each cluster of homologous genes (Group/Ungroup the homologs options); (2) color network nodes using either heatmap or blue-red gradient schemes according to evolutionary characteristics of genes (cluster memberships or evolutionary indices).

### Analysis of gene evolutionary indices

**Fig. 2:**
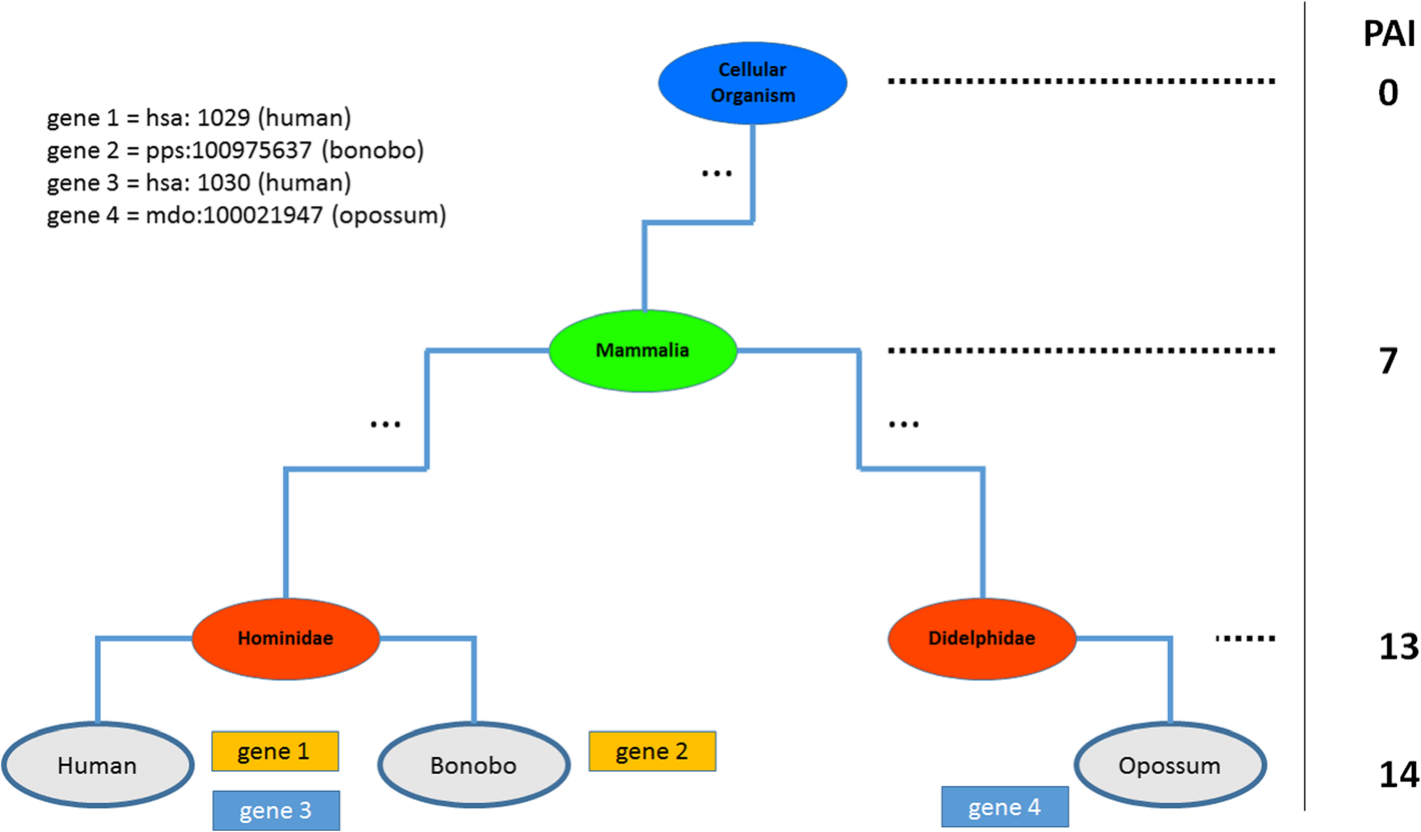
PAI calculation. Part of the taxonomic tree illustrating the PAI calculation. For gene 1, the only ortholog gene was found in Bonobo (gene 2). It means the evolutionary age of gene1 and gene 2 is 13 (“Hominidae” – young genes). Contrariwise, for gene 3 we found ortholog even in opossum (gene 4). It means that genes diverged on Mammalia stage, so the PAI is equal to 7 (“Mammalia” – “moderate age” genes)

The Orthoscape calculates two evolutionary characteristics of genes. The first characteristic is the phylostratigraphic age index (PAI). The PAI indicates the “evolutionary age” of a gene [30, 31]. To calculate the PAI, the Orthoscape uses KEGG Organisms database for taxonomic trees. It performs a search of orthologous genes (using sequence similarity thresholds mentioned above), populates the tree of species these genes belong to and then analyses the resulting tree. Ranged between 0 and N (where N is a number of phylums between root and a species in the taxonomic tree; for instance, *N* = 14 for human), this index shows the level of the last common ancestor node in the whole taxonomic tree, containing at least one species from the list of species possessing orthologs of a gene under analysis (Fig. 2). Consequently, the “Cellular Organisms” node (root) has PAI = 0, “Eukaryota” node has PAI = 1, etc.; “Homo” node has PAI =14).

The second characteristic is the Divergence Index (Ka/Ks index, DI) of a gene [32, 33]. It indicates the influence of natural selection on gene evolution. To calculate the DI, Orthoscape extracts nucleotide sequences and amino acid sequences of a gene/protein under analysis and its nearest ortholog (from the closest species). Amino acid sequences are retrieved via KEGG REST API and then aligned using the Needleman–Wunsch algorithm [34] using NW-align code (http://zhanglab.ccmb.med.umich.edu/NW-align/). The codon alignment is obtained using the protein alignment; Ka/Ks ratio is calculated using Java code from the program Ka/Ks calculator [35] (http://kakscalculator.fumba.me/index.jsp). In general, the DI may be calculated through the analysis of any orthologs found, however it is recommended to use the closest taxa for DI calculation [30]. In Orthoscape, the user may specify the maximum distance between the reference species and other nodes on the species tree. Besides, for human gene networks we provide an additional option that allows explicit specifying of the closest species – Pan troglodytes (chimpanzee), Pongo abelii (sumatran orangutan), Pan Paniscus (bonobo) and working with orthologs belonging to these only organisms.

Additionally, Orthoscape reports phylogenetic profile [36] for each gene in the network. This is a table with columns corresponding to genes represented in the metabolic network, and rows corresponding to organisms which genomes contain at least one ortholog of the network genes. The cell in column *i* and row *j* contain ‘+’ if in the organism *j* exists at least one ortholog of gene *i*, or ‘-‘otherwise.

### Report generator

Orthoscape generates reports with all calculated characteristics, indices, statistics etc. obtained for one or several gene networks. Report is generated in HTML format and also includes directory with additional image files. Report contains PAI histograms and tables of genes sorted by DI. Analyzing PAI histograms, the Orthoscape reports “average evolutionary age” of a gene network and/or gene set. First of all, it concerns to the mean and median PAI values for all genes in network/set. Once the user tried different SW-Score and identity parameters in his/her analysis, all of these attempts are included into the report.

Orthoscape provides both Gene Set and Network PAI statistics. The first one is calculated with the simple formula:

$$ \mathrm{Gene}\ \mathrm{Set}\ \mathrm{P}\mathrm{A}\mathrm{I} = \frac{{\displaystyle {\sum}_{\mathrm{genes}}}PAI}{N} $$

Where *N* is the number of genes. Thus, if all genes are ancient (PAI = “Cellular organisms”), then the statistics value would be 0. If all genes are “young” (PAI = “Homo”), then the statistics value would be 14. Therefore, the more is the mean PAI value, the “younger” genes are in the gene set.

Network PAI statistics additionally takes into account the network topology, more specifically connectivity of nodes (i.e., their degrees). For example, if one node is connected with two edges, its degree would be 2, if it has no connections, the degree would be 0 etc. The modified formula is used:

$$ \mathrm{Network}\ \mathrm{P}\mathrm{A}\mathrm{I} = \frac{{\displaystyle {\sum}_{\mathrm{nodes}}}\left(PAI*d\right)}{2N} $$

Where *d* is the node degree. It makes highly connected nodes, which we believe are more important in gene networks functioning than lowly connected ones to have higher contribution to the “evolutionary age” of a gene network.

There are also median, oldest and youngest statistics for PAI values in a network. Finally, the total number of orthologs analyzed is reported too. An example of statistical analysis from report is shown below in Results section.

### Visualization of results

Analyzed network may be colored using two schemes: (1) Heatmap scheme colors “young” genes in red and “old” in blue. Intermediate colors are yellow, green, cyan; (2) Blue-white-red gradient scheme. Each style may be used for PAI, DI and homologous groups.

## Results and discussion

As an example of the Orthoscape application, we have analyzed two pathways from KEGG database related to steroid metabolism: steroid biosynthesis pathway and steroid hormone biosynthesis pathway.

Steroids, such as cholesterol, are synthesized in almost all eukaryotic cells, which use these triterpenoid lipids to control the fluidity and flexibility of their cell membranes. Sterols also play a key role in such eukaryotic features as phagocytosis. In KEGG, steroid biosynthesis pathway (ko00100) follows the terpenoid backbone biosynthesis pathway and uses farnesyl diphosphate as input metabolite. A number of sterols produced as a result of this pathway: zymosterol, fecosterol, episterol, ergosterol and others. Interestingly, few bacteria can synthesize sterols [37], however, phylogenetic analysis demonstrated that they likely acquired homologs of enzymes of the sterol pathway via ancient horizontal gene transfer from eukaryotes [38].

An important metabolite produced within the sterol biosynthesis pathway is cholesterol, an essential structural component of all animal cell membranes and precursor for the biosynthesis of steroid hormones [39, 40]. Five major classes of steroid hormones include testosterone, progesterone and estradiol, which are known as sex-steroids, and cortisol/corticosterone and aldosterone, which are referred to as corticosteroids [39]. Steroid hormones are synthesized from cholesterol through a common precursor steroid, pregnenolone, which is formed by the enzymatic cleavage of a 6-carbon side-chain of the 27- carbon cholesterol molecule by the cytochrome P450 side-chain cleavage enzyme [39]. Their biosynthesis is described in KEGG by steroid hormone biosynthesis pathway (ko00140).

**Fig. 3:**
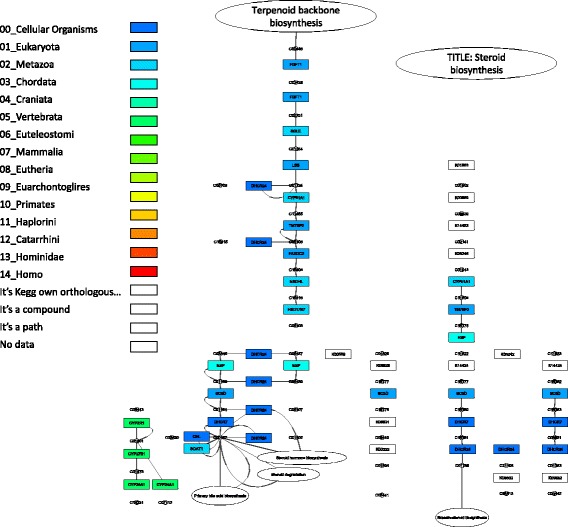
KEGG steroid biosynthesis pathway. Visualization of the KEGG steroid biosynthesis pathway (ko00100) by the Orthoscape application using PAI heatmap color scheme. Gene node colors correspond to PAI values from smaller (PAI = 0, older genes, dark blue color) to larger (PAI = 5, young genes, green color)

**Fig. 4:**
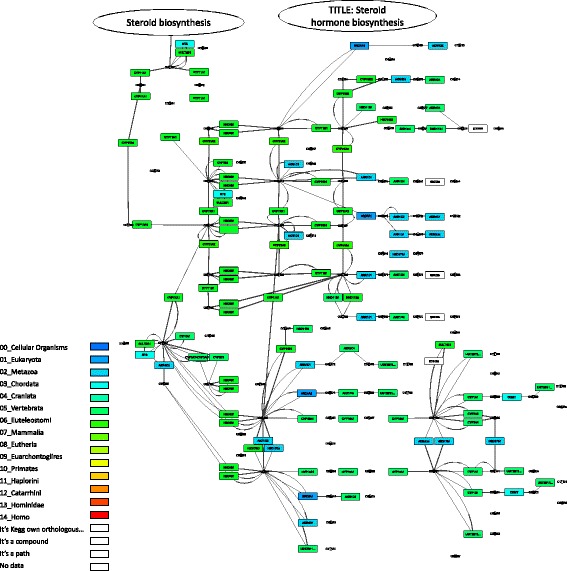
KEGG steroid hormone biosynthesis pathway. Visualization of the KEGG steroid hormone biosynthesis pathway (ko00140) by the Orthoscape application using PAI heatmap color scheme. Gene node colors correspond to PAI values from smaller (PAI = 1, older genes, blue color) to larger (PAI = 7, young genes, green color)

**Fig. 5:**
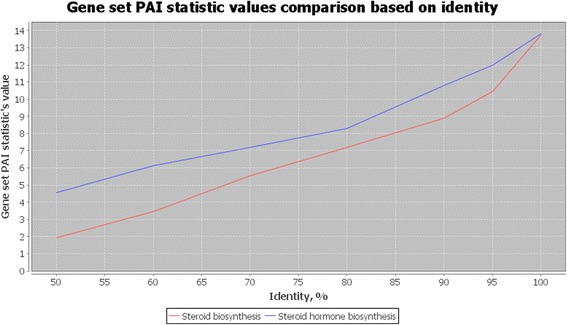
Overall PAI comparison for steroid pathways. Comparison of the dependence of the PAI indices (Y axis) for steroid biosynthesis pathway (red line) and steroid hormone biosynthesis pathway (blue line) with respect to identity threshold for gene orthology detection (X axis)

Visualization of these two pathways is shown in Figs. 3 and 4. The visualization demonstrates the PAI for each gene in the metabolic network graph, from blue (small PAI, older genes) to green (large PAI, younger genes). For example, for delta24-sterol reductase (gene ID DHCR24, EC:1.3.1.72, 1.3.1.-), which participates in sterol biosynthesis pathway (Fig. 3), the PAI is equal to 0 (‘Cellular Organisms’ taxonomic group, dark blue color). For vitamin D 25-hydroxylase (gene ID CYP24A1, EC:1.14.14.24) in the same pathway (Fig. 3), the PAI value is 5 (‘Vertebrata’ taxonomic group, green color). It could be seen from the comparison of these two pathway diagrams (Figs. 3 and 4) that the steroid hormone biosynthesis pathway contains larger fraction of younger genes as compared to the steroid biosynthesis pathway. It is demonstrated by comparison of the gene set PAI values for these two pathways for different orthology detection thresholds (Fig. 5). The PAI values for steroid hormone biosynthesis pathway are higher at all thresholds except in the case of identity is equal to 1. These results demonstrate that most genes for this pathway diverged from their ancestors and acquired their new function later as compared to genes from steroid biosynthesis pathway.

The obtained results of comparison of the gene ‘ages’ for the above pathways are consistent with the current knowledge of the eukaryotic evolution. Steroids participate in the formation of membranes, which are basal cellular structures in eukaryotes. Thus, sterol biosynthesis is a fundamental feature of eukaryotic cells and it is generally accepted that the pathway of sterol biosynthesis appeared after the emergence of oxygenic photosynthesis and the oxygenation of the atmosphere and oceans (between 2.7 and 2.4 Ga) [41].

Steroid hormones regulate diverse physiological functions such as reproduction, blood salt balance, maintenance of secondary sexual characteristics, response to stress, neuronal function, various metabolic processes [40] and response to environmental factors [42]. In mammals, they are synthesized in a specific set of organs: ovary (granulosa cells, luteal cells), testis (Leydig cells), adrenal gland (zona glomerulosa and zona reticularis cells), placenta and brain (neurons, glial and Purkinje cells) [40]. The analysis of the evolution of the steroid hormone receptors demonstrates, that they are common to vertebrates [43], but might have originated before the divergence of vertebrates, suggesting the ancient origin of some steroid hormone systems (as was shown for the estrogen signaling [44]). It should be noted, however, that hormones are the important source of the organism’s phenotypic plasticity [45]. For example, placenta, the evolutionary innovation of mammals, demonstrates amazing diversity [46]. These phenotypic innovations should require evolutionary changes in the hormone biosynthesis pathway, for example, increasing its complexity to produce different types of hormones. This is the likely reason for the relatively modest age of the genes involved in the steroid hormone biosynthesis.

**Fig. 6:**
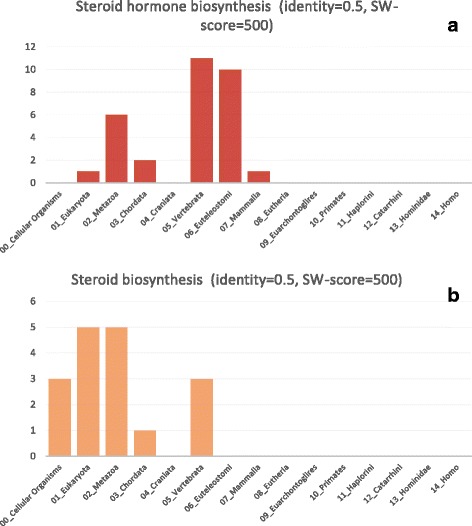
PAI comparison for steroid pathways with identity 0.5. Distribution of PAI among genes in networks steroid hormone biosynthesis (**a**) and steroid biosynthesis (**b**) networks

Figure 6 shows the histograms for the distribution of PAI values for genes from both networks (SW-Score = 500, identity = 0.5, 0 domains). On histograms, one could see that steroid hormone biosynthesis pathway (Fig. 6a) includes larger fraction of genes with PAI from 5 (‘Vertebrata’) to 7 (‘Mammalia’) as compared to steroid biosynthesis pathway (Fig. 6b). Orthoscape reports the following statistics for steroid biosynthesis pathway: Gene set PAI = 4.548; Network PAI = 4.828; Median taxon = Vertebrata; Oldest taxon = Eukaryota; Youngest taxon = Mammalia. Values for steroid biosynthesis pathway were as follows: Gene set PAI = 1.941; Network PAI = 1.4; Median taxon = Metazoa; Oldest taxon = Cellular Organisms; Youngest taxon = Vertebrata.

Note, that data from Figs. 5 and 6 and PAI statistics could be found in HTML reports output by Orthoscape. Global report (PAI values dependence on the sequence similarity thresholds for a set of networks, see Fig. 5) and networks specific reports (PAI distribution histograms, see Fig. 6) could be navigated using hyperlinks. The original data for the plots in text format and links to networks information at the KEGG web-site are provided.

**Fig. 7:**
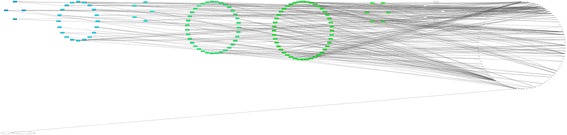
Attribute layouting (by PAI) example. Visualization of the KEGG steroid hormone biosynthesis pathway using layout based on the PAI similarity. In this layout scheme, genes with identical PAI values are located on the same circle

Figure 7 demonstrates the additional layout scheme for the networks based on PAI identity. This type of network layout shown for the steroid hormone biosynthesis pathway: genes are located on the circles corresponding to different PAI values (genes with identical PAI are located on the same circle); non-gene nodes are located on the separate circles. It helps to identify genes with the same “age” easily.

**Fig. 8:**
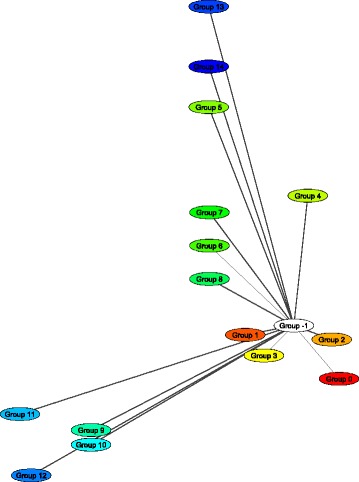
Grouping to homology clusters example. Visualization of the KEGG steroid biosynthesis pathway using layout based on the homology cluster membership (all homologous genes are joined in the same nodes)

The gene network layout based on the grouping of genes with respect to their membership in different homology clusters is demonstrated in (Fig. 8) for the steroid biosynthesis network. There were 14 homologous clusters found for this network. By expanding the node on this type layout user can obtain the list of genes in the cluster. Detailed report generated by the Orthoscape on this comparison is presented in the Additional file 3.

## Conclusion

In summary, Orthoscape application is a tool for analysis of gene networks/sets. It allows to search for homologs (orthologs and/or paralogs), to perform phylostratigraphic analysis of genes and to investigate the divergence. User is aided in better understand of evolution of genes and their (sub)networks under selective pressure. At each step of data analysis, Orthoscape also provides for convenient visualization and data manipulation.

## Additional files

Additional file 1: Orthoscape-1.0. The plugin itself. (JAR 105 kb)

Additional file 2: Orthoscape manual. The manual with basics of Orthoscape. (DOCX 133 kb)

Additional file 3: Reports. 7z archive containing the Orthoscape reports example. (7Z 381 kb)

## Abbreviations

API: Application program interface
DI: Divergence index
PAI: Phylostratigraphic age index
REST: Representational state transfer
