# Supplementary material for: Orthoscape: a cytoscape application for grouping and visualization KEGG based gene networks by taxonomy and homology principles
Source: BMC Bioinformatics. 2017 Jan 27;18(Suppl 1):1–9. doi: 10.1186/s12859-016-1427-5 (PMC5333177; doi:10.1186/s12859-016-1427-5)
Supplement: Supplementary file 2 — Orthoscape manual. The manual with basics of Orthoscape. (DOCX 133 kb) [file 12859_2016_1427_MOESM2_ESM.docx]

This file contains the basic instructions how to use Orthoscape application.

# Installation

First, you have to put “jar” file into the Cytoscape application’s directory. It can be founded in the path like: C:\Users\”YourUserName”\CytoscapeConfiguration\3\apps\installed in windows OS.

Than you have to download any one of the utility applications (CyKEGGParser, GeneMANIA, CyPath2). They are the way to get input network. You can find these applications in the Cytoscape app Store.

We recommend to use CyKEGGParser to try Orthoscape first time. It greatly works with our application without any convertations. You can download any network using CyKEGGParser online downloading ability.

# Creating network

You can create your own networks. There are 4 important columns in 2 important tables. If you want to use your own networks, you have to create next columns:

1) Node Table column **“name”**. It should contains the organism type and the gene Entrez ID separated by “:” (for example hsa:1029 should be typed in the “name” ).

2) Node Table column **“Type”**. It should contains the type of the element. It’s “gene” for genes (the only type we analyze now).

3) Network Table column **“title”**. It should contains the name of the network.

4) Network Table column **“org”**. It should contains the KEGG acronym of the specie (for example “hsa” for Homo Sapiens).

The best way to understand it is to use CyKeggParser to import any network as the template and to change data in the accessible network.

# General workflow


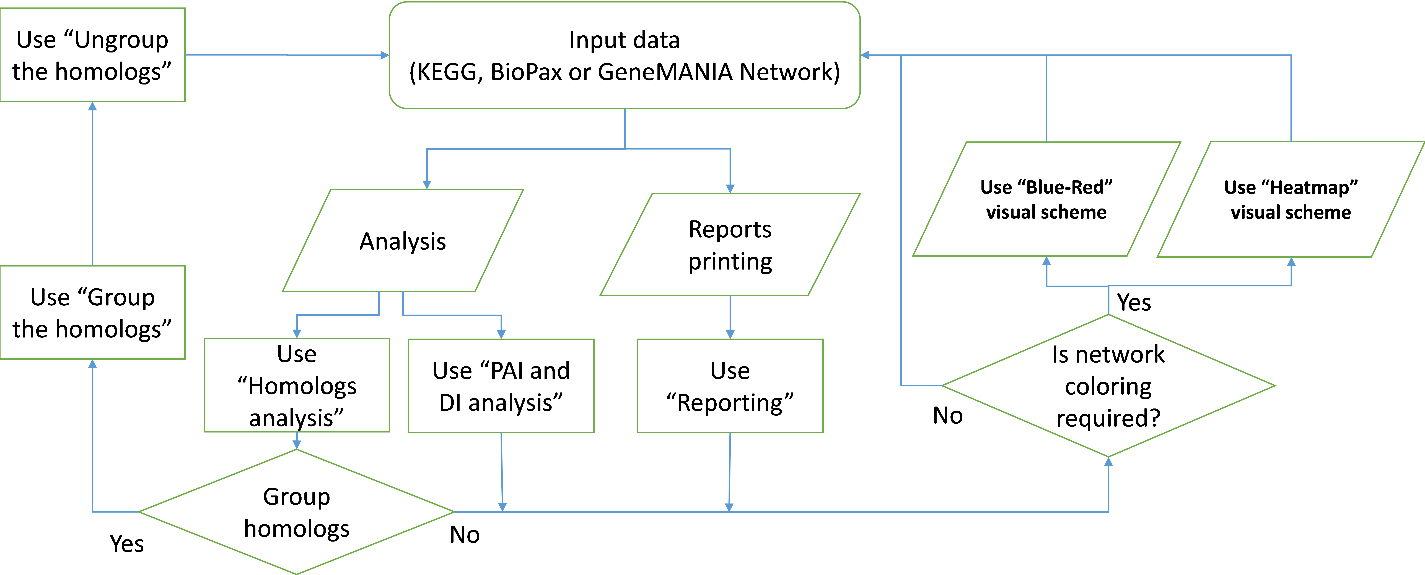


If you have a network to analyze then the scheme of your work will be:

There are five global options in the application:

1) **Converting** (Convert GeneMANIA network/Convert CyPath2 network).

2) **Working** (Homologs analysis, PAI and DI analysis).

3) **Grouping** (Group the homologs/Ungroup the homologs).

4) **Coloring** (Blue-Red/Heatmap -> PAI Based/DI Based/Homology Based).

5) **Reporting**.

## Converting

Converting allows to convert networks obtained by GeneMANIA or CyPath2 applications to accessible format. You should use this option since you obtained the network from GeneMANIA or CyPath2 applications.

## Working

Working allows to analyze homology relationships and evolutionary indices. There are two ways to work with your data:

### “Homologs analysis”


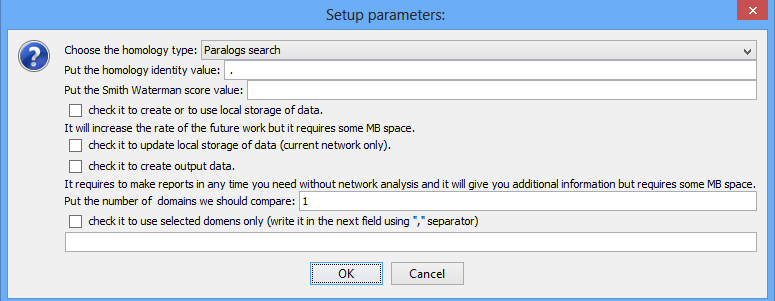


Here you can analyze genes and divide them into homology clusters. The “Paralogs search” using by default but you can change it to ”Orthologs search” if you are interested in analyzing genes from different species. There are some steps to be done:

1) You should fill the identity and SW-Score fields by the threshold valued (the minimum value enough to accept homologs form KEGG database).

2) You should check from 0 to 3 checkboxes. First checkbox allows you to created local base for every important data to greatly decrease the time of working with same network in the future. Second checkbox allows you to update local base. If you have network data in the base but don’t want to use them (for example you doesn’t work with this network a lot of time and your data probably out of date) you should check second checkbox. Third checkbox allows you to create additional output data (domains information for genes and their homologs, full taxonomy rows for gene and their orthologs, etc.) and statistic files (they will be used to created reports later). This information doesn’t influence to the time of future work.

3) You should put the number of domains, which homolog from base must contains to be accessed like homolog (these domains must be same to homolog and gene. If not then homolog discarded) or wright concrete domains if you want to analyze only genes and homologs with these domains.

After installing your option, press OK to start the work. If you checked any of base creating checkboxes then the program will ask you to choose the base directory. After the program will finish the work, you will see “Homology Cluster” column. If the values in two different rows are same then the genes placed to one cluster. There are one or more homologous genes for every gene in cluster. You can use coloring and attribute layouting to represent the results on network.

### “PAI and DI analysis”


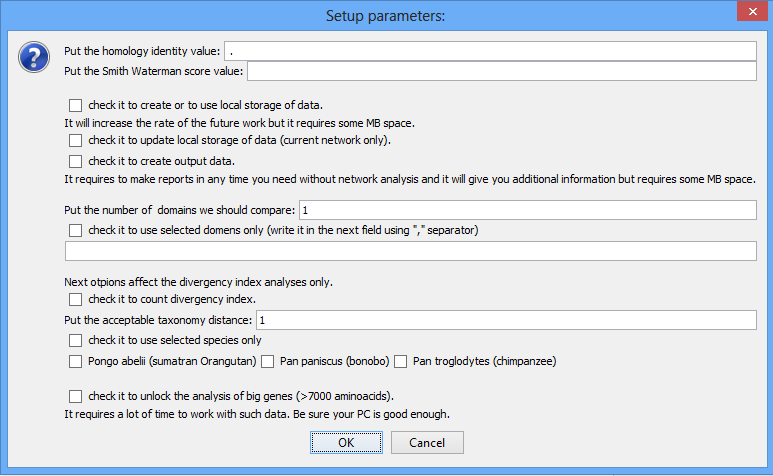


There are some new options. They are relevant for DI analysis only. First is the checkbox to turn DI analysis on. You have to check it if you want to make DI analysis.

Second parameter is difference threshold. It represents the maximum accessible taxonomy difference rate between gene’s organism and ortholog’s organism (for example, if we analyze Homo Sapiens (Eukaryota/…/Hominida/Homo) then difference rate is equal to 1 for any Hominida ortholog specie). The DI will be counted if the difference rate <= difference threshold. You also can use only three nearby to Human species. Check the checkbox and choose the species you want to analyze.

Finally, you can unlock the analysis of big genes. Some genes like TTN contains thousands of amino acids. It makes the Needleman–Wunsch alignment very time consuming. Be sure you have a lot of time and good enough PC to analyze such genes.

**Note:** All previous parameters are actual only if you want to analyze DI. If PAI enough for you then these options can be missed. Big genes analysis works fine for PAI without any dependence of last checkbox.

After the program will finish the work, you will see five new columns:

1) “PAI” shows farther to taxonomy tree root phylum overall for gene and every ortholog of this gene.

2) “PAI Power” shows the number of orthologs analyzed to obtain PAI.

3) Node Degree shows the number of edges actual to this node.

4) DI Average shows the average value obtained after counting DI’s between gene and every ortholog.

5) DI Variance shows the variance value obtained after counting DI’s between gene and every ortholog.

## Grouping

Grouping allows to group and ungroup homologs into clusters. Be sure to use “ungroup the homologs” every time after using “group the homologs” if you want to count anything else in this network.

## Coloring

Coloring allows to visualize your network based on one of the visual styles and one of the input columns.

## Reporting

Reporting allows to create reports. Every time you used “Output data” checkbox the program saved statistics files. Reporting option allows you to create html reports and PNG pictures based on these files.
